# From ELF to Compressibility in Solids

**DOI:** 10.3390/ijms16048151

**Published:** 2015-04-13

**Authors:** Julia Contreras-García, Miriam Marqués, José Manuel Menéndez, José Manuel Recio

**Affiliations:** 1 MALTA-Consolider Team Universidad de Oviedo, E-33006 Oviedo, Spain; E-Mails: miriam.marques@uva.es (M.M.); jmenendezmontes@gmail.com (J.M.M.); jmrecio@uniovi.es (J.M.R.); 2 Université Pierre et Marie Curie, Univ Paris 06, UMR 7616, Laboratoire de Chimie Théorique, F-75005 Paris, France; 3 Centre National de la Recherche Scientifique, Unité Mixte de Recherche 7616, Laboratoire de Chimie Théorique, F-75005 Paris, France; 4 Departamento de Química Física y Analítica, Universidad de Oviedo, E-33006 Oviedo, Spain; 5 Departamento de Física Teórica, Universidad de Valladolid, E-47011 Valladolid, Spain

**Keywords:** ELF, compressibility, energy models

## Abstract

Understanding the electronic nature of materials’ compressibility has always been a major issue behind tabulation and rationalization of bulk moduli. This is especially because this understanding is one of the main approaches to the design and proposal of new materials with a desired (e.g., ultralow) compressibility. It is well recognized that the softest part of the solid will be the one responsible for its compression at the first place. In chemical terms, this means that the valence will suffer the main consequences of pressurization. It is desirable to understand this response to pressure in terms of the valence properties (charge, volume, *etc.*). One of the possible approaches is to consider models of electronic separability, such as the bond charge model (BCM), which provides insight into the cohesion of covalent crystals in analogy with the classical ionic model. However, this model relies on empirical parametrization of bond and lone pair properties. In this contribution, we have coupled electron localization function (ELF) *ab initio* data with the bond charge model developed by Parr in order to analyze solid state compressibility from first principles and moreover, to derive general trends and shed light upon superhard behavior.

## 1. Introduction

A thorough understanding of many important electronic and mechanical properties of a crystalline material does not require the knowledge of the total energy of the solid, but just the energetics of its bonding network. Many phenomenological approaches have been developed to illustrate the advantage of this view [1]. These so-called bond energy models are useful for properties prediction and have become rather popular in the last years as trusty guides for material design. Specifically to the field of materials strength, Gilman’s book [1] collects a number of valuable correlations between a variety of observable elastic moduli of different type of solids and electronic densities coming only from the active electrons of the valence space. Based on the ideas developed by Gilman [2], Gao *et al.* “initialized a link” between macroscopic observables and first principles calculations with the aim to evaluate the indentation hardness of multicomponent crystals [3]. Li *et al.* resorted to bond electronegativity in order to evaluate the bond energy and thus figure out the most stable bond combination for hard materials [4]. In conjunction with evolutionary algorithms [5], Li’s model is theoretically generalized by Lyakhov and Oganov making use of the bond-valence model and graph theory [6].

Inherent to all these semi-empirical models, there are still elusive concepts to quantum-mechanical formalisms, as ionicity/covalent scales, electronegativities, covalent radii, *etc.* Efforts from the theoretical chemistry community have been devoted to reformulate these concepts from first principles. As an example, we can cite recent reformulations of electronegativity [7,8,9]. These chemical concepts do not derive directly from wavefunction analysis, but a posteriori models are needed to extract them. The bondon [10] and the reformulation of chemical bonding as a quantum condensate are some of the formulations that have been recently proposed [11]. Another approach that has gained many followers is the topological formalism. This approach divides three-dimensional space into chemically meaningful disjoint regions following the gradient of a scalar function, such as the electron density [12,13] or the electron localization function (ELF) [14,15]. Since we are interested in valence properties, our function of choice is the latter one. ELF is able to recover Lewis entities, such as bonds and lone pairs and quantify their properties (volume, charge, *etc.*). Due to its direct link with Lewis theory, we have chosen the model by Borkman and Parr [16] as our starting point to merge topology and bond energy models.

Briefly, our study is based on the success of the bond charge model [16,17,18] in predicting molecular properties, and the ability of the electron localization function [14,15] to provide the required parameters for its free from empirical data application. The bulk modulus (inverse of the compressibility) will be the focus of this first principles-like extension of BCM to the solid state.

The remainder of the paper is organized as follows. Firstly, the original definition of the bond charge model for homo and heteronuclear binary molecules and covalent solids will be presented. Secondly, the computational details for the calculations performed over a broad group of diamond-type and zinc-blende-type solids from the IV, III–V and II–VI groups are given. In Section 3, the basic parameters of this *ab-initio* BCM (hereafter, NEWBCM) are derived from first principles. The ability of this model to predict solid state properties (compressibility) will be tested against experimental data in Section 4. At the end of the section, this new insight into macroscopic properties will be used to understand macro-micro requirements for superhard materials. The article finishes with the main conclusions and guidelines for prospective work.

## 2. Theoretical Backgroud

In this section, the two approaches to be merged are introduced: the bond charge model and the ELF topological formalism.

### 2.1. The Bond Charge Model: Original Definition

#### 2.1.1. Homo and Heteronuclear Diatomic Molecules

**Figure 1:**
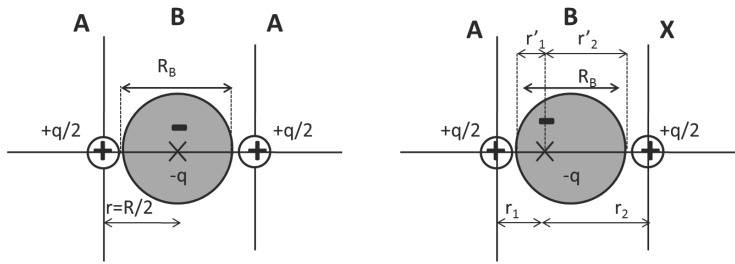
Bond charge model for homonuclear (**left**) and heteronuclear (**right**) molecules, *A*_2_ and *AX*, respectively. The meaning of the main parameters that are needed in each case is shown in the figures: *q* and *R_B_* stand for neutral homonuclear molecules as *q*, *δ*, *r*_1_/*r*_2_ and *R_B_* do for heteronuclear ones; B label stands for the bond (see text for more details). $r_{1}^{\prime }$ and $r_{2}^{\prime }$ are also shown for clarity.

Within the BCM formalism [16], a homonuclear diatomic molecule (*A*_2_) can be approximated by two cores holding a *q*/2 positive charge each, and a bond charge in between them, -*q*, that moves freely along a bond length *R_B_* = *νR* which is a fraction (*ν≤* 1) of the interatomic distance *R* (see Figure 1 (left)). The energy associated to this model at distances close to the equilibrium (*R* ≃ *R_e_*) is given in atomic units by:

(1) $$E(q,R)=E_{0}+E_{1}+E_{2}=2E_{A}-\frac{Cq^{2}}{R}+\frac{D\prime q}{R_{B}^{2}}$$

where *E*_0_ represents the core energy (which equals to 2*E_A_* for an *A*_2_ molecule), *E*_1_ refers to the coulombic attractive interactions and *E*_2_ is related to the kinetic energy of the bond electrons approximated as particles in a box of length *R_B _*. The constants, which are system independent, are then given by (see [16] for details):

(2) $$C=\frac{7}{4},\;\;D^{\prime }=\frac{\pi^{2}}{2m}$$

and the parameters *q* and *ν* are inferred from empirical measures of the harmonic force constant, *k_e_*, and the equilibrium distance, *R_e_*.

In order to adapt this model to heteronuclear diatomic molecules, *AX*, or even to homonuclear molecules in an asymmetric surrounding, two considerations have to be taken into account. On the one hand, the polarity of the bond clearly influences the situation of the bond charge (Figure 1 (right)), being *r*_1_ and *r*_2_ the distances to its position from atoms *A* and *X*, respectively. On the other hand, the positive charge lying on each of the two nuclei will be different from $\frac{1}{2}$*q* by $\frac{1}{2}$*δ* and $-\frac{1}{2}$*δ* [18]. The changes induced by the electronegativity difference, ${∆}_{\chi =\chi_{A}-\chi_{X}}$, in *E*_2_ are negligible, but the bond charge shift gives rise to noticeable changes in the coulombic interactions:

(3) $$E_{1}=\frac{q^{2}}{2}\left[\frac{\left(1-\delta^{2}\right)}{2R}-\frac{\left(1+\delta \right)}{r_{1}}-\frac{\left(1-\delta \right)}{r_{2}}\right]$$

so that the total energy reads as follows:

(4) $$E=E_{A}(q,\delta )+E_{X}(q,\delta )+\frac{q^{2}}{2}[\frac{\left(1-\delta^{2}\right)}{2R}-\frac{\left(1+\delta \right)}{r_{1}}-\frac{\left(1-\delta \right)}{r_{2}}]+\frac{D^{\prime }q}{R_{B}^{2}}$$

The determination of the model parameters, *q*, *δ*, *r*_1_/*r*_2_ and *R_B_* has become a matter of choice. Probably, the most extended method has been to take into account the electronegativity of the atoms as shown by Parr *et al.* [19,20].

#### 2.1.2. Binary Covalent Solids

The model by Parr and Borkman was extended by Martin [21] to describe the energy of covalent crystals. For simplicity, we consider a zinc-blende-type solid, *AX*, with a formula unit *AXB*_4_ accounting for the four-fold coordination of the two elements in the unit cell, *B* represents the bond entity. According to BCM, the energy of the covalent solid per couple of atoms *AX* is given by

(5) $$E_{AX}\;=\;E_{A}(q_{B},\delta )+E_{X}(q_{B},\delta )+E_{B}(q_{B},\delta ,R_{B})$$

(6) $$E_{B}\;=\;-\frac{Mq_{B}^{2}}{R_{B}}+\frac{4D^{\prime }q_{B}}{R_{B}^{2}}$$

where *M* is the Madelung constant for these lattices with charges 2*q_B _*(1 + *δ*) and 2*q_B _*(1 *−* *δ*) at *A* and *X* atomic sites, and *−q_B_* at each bond position, as dictated by the multiplicity and the charge neutrality condition. We should recall that *E_A_* and *E_X_* account for the energy of the corresponding core electrons not involved in the bonding, and, in practice, are independent of geometrical parameters. The greatest modification introduced in the application of BCM to solids is the presence of an infinite sum, accounting for all *A*, *X*, and *B* charged species, which is subsumed into the Madelung constant. It it also important to highlight that due to the inclusion of the bond charge in the summation, this Madelung constant is system dependant since the position of the bond charge will not correspond to a special Wyckoff position (invariable) except for homonuclear solids. The Madelung constants have been calculated thanks to the environ code [22] and are collected in Table 1.

**Table 1:** Parameters of the non-empirical new bond charge model (NEWBCM), [*q_B_*, *R_B_*, *r*_1_/*r*_2_], for a broad group of diamond-type and zinc-blende-type solids from the IV, III–V and II–VI groups. *r*_1_(*r*_2_) stands for the distance between the charge, *q*, and the nucleus *A*(*X*). $r_{1}^{\prime }(r_{2}^{\prime })$ stands for the part of *R_B_* belonging to *r*_1_(*r*_2_), *i.e.*, $r_{1}^{\prime }=r_{1}-r_{A}$ and $r_{2}^{\prime }=r_{2}-r_{X}$, where *r_A_* is the core size of atom *A* and *r_X_* is the core size of atom *X*. *M* and *E_B_* stand for the Madelung constant and the bonding energy of the crystal, respectively. The cell parameter, *a*, and the electronegativity difference [23], ${∆}_{\chi =\chi_{X}-\chi_{A}}$, are also shown for reference. Lengths are in Å, charges in electrons, energy in hartrees.

AX	*a*	∆*_χ_*	*r*_1_*/r*_2_	$r_{1}^{\prime }/r_{2}^{\prime }$	*R_B_*	*q_B_*	*M*	*E_B_*	*δ*
C	3.557	0.0	1.00	1.00	0.938	1.950	10.856	*−*11.644	0.000
Si	5.431	0.0	1.00	1.00	1.132	1.950	8.577	*−*7.623	0.000
Ge	5.658	0.0	1.00	1.00	1.003	2.100	7.414	*−*8.625	0.000
SiC	4.360	0.7	1.17	0.75	0.985	1.950	9.356	*−*9.556	0.000
BN	3.616	1.0	1.07	0.86	0.936	1.975	11.006	*−*12.136	0.241
BP	4.538	0.1	0.96	1.21	1.050	2.000	9.662	*−*9.739	0.250
BAs	4.777	0.0	0.70	0.82	1.023	2.050	8.711	*−*9.468	0.268
BSb	5.156	0.1	0.62	0.88	0.999	1.988	8.554	*−*8.954	0.245
AlP	5.464	0.6	1.14	0.97	1.122	1.975	8.925	*−*8.206	0.266
AlAs	5.661	0.5	0.96	0.83	1.080	2.025	8.028	*−*8.068	0.284
AlSb	6.136	0.4	0.81	0.77	1.093	1.963	7.304	*−*6.810	0.261
GaAs	5.653	0.4	1.19	1.18	0.992	2.100	7.662	*−*9.008	0.238
GaSb	6.096	0.3	1.00	1.12	0.986	2.038	6.764	*−*7.538	0.215
InP	5.869	0.4	1.64	1.38	1.012	1.970	8.494	*−*8.615	0.269
InAs	6.058	0.3	1.35	1.11	0.958	2.020	7.268	*−*8.187	0.287
CdS	5.818	0.8	1.89	1.48	0.976	1.975	10.016	*−*10.512	0.494
CdSe	6.077	0.7	1.56	1.27	0.938	2.025	8.591	*−*9.697	0.506
ZnS	5.410	0.9	1.67	1.50	1.001	2.025	10.130	*−*10.976	0.457
ZnSe	5.668	0.8	1.37	1.30	0.970	2.075	8.631	*−*10.134	0.470
ZnTe	6.104	0.5	1.15	1.25	0.971	2.038	7.415	*−*8.389	0.460

From Equation (6), the bond energy of a zinc-blende-type lattice can be finally expressed within the framework of BCM as:

(7) $$E_{B}=-\frac{Mq_{B}^{2}}{R_{B}}+\frac{4D^{\prime }q_{B}}{R_{B}^{2}}$$

It is interesting to highlight here that this expression is similar to the classical Madelung energy expression for ionic crystals. However, in our case the equation is done in terms of Lewis entities (cores, bonds) instead of ions. The expression will then hold as long as the orthogonality of these entities holds too. In solids such as diamond, where for example, good localized orbitals can be found, Equation (7) will give an accurate description of the bonding energy in the crystal. However, as the localized picture fails (e.g., down the periodic table), we should expect the fits to worsen. This equation will progressively fail as we go down the periodic table or move towards metallic solids, due to a less obvious separability.

If we reasonably assume that (*∂E_B_*/*∂_R_*) ≃ 0 at equilibrium then

(8) $${\left(\frac{\partial E_{B}}{\partial R_{B}}\right)}_{R=R_{e}}=\frac{Mq_{B}^{2}}{R_{B}^{2}}-\frac{8D^{\prime }q_{B}}{R_{B}^{3}}=0$$

where we have assumed that the ratio *r*_1_/*r*_2_ remains constant upon compression, not introducing changes in *M* (see discussion in last section and Figure 5). It is very important to notice that all the parameters have to be evaluated at equilibrium. We then have for the constant *M*:

(9) $$M=\frac{8D^{\prime }}{q_{B}R_{B}}$$

Hence, the ground state bond energy at equilibrium is given by:

(10) $$E_{B,eq}=-\frac{4D^{\prime }q_{B}}{R_{B}^{2}\;}=-\frac{Mq_{B}^{2}}{2R_{B}}$$

where the negative value reflects the stabilization introduced by the chemical bond (see Table 1). This equation, just like in the original bond charge model [16,18], is a manifestation of the virial theorem. Upon substitution of Equation (9) in Equation (7), it can be seen that at equilibrium, the electrostatic interaction is twice as big as the kinetic energy term.

### 2.2. ELF Topology

The Electron Localization Function, ELF, was originally designed by Becke and Edgecombe to identify “localized electronic groups in atomic and molecular systems” [14]. Several interpretations have been given to this function. For example, ELF has been generalized and reinterpreted in the light of Markovian processes [24,25]. A very intuitive interpretation was given by Savin [15], according to which the ELF core, χ, can be understood as a local measure of the excess of local kinetic energy of electrons due to the Pauli principle, *t_p_*:

(11) $$\chi_{\sigma }(r)=\frac{t_{p}(\vec{r})}{t_{HEG}(\vec{r})}$$

relative to the homogeneous electron gas kinetic energy density, $t_{HEG}(\vec{r})=\frac{3}{5}{\left(6\pi^{2}\right)}^{2/3}\rho_{\sigma }^{5/3}(\vec{r})$. *t_p_* is calculated as the difference between the definite positive kinetic energy, $t(\vec{r})$, and the von Weizsäcker kinetic energy functional:

(12) $$t_{p}(\vec{r})=t(\vec{r})-\frac{1}{4}\frac{|\rho_{\sigma }(\vec{r}){|}^{2}}{\rho_{\sigma }(\vec{r})}$$

ELF values are confined in the [0,1] range by a Lorentzian transformation which facilitates its interpretation:

(13) $$ELF=\frac{1}{1+\chi 2}$$

ELF maxima appear associated with localized electron pairs, such as bonds, lone pairs and atomic shells, thus matching the Valence Shell Electron Pair Repulsion [26] model. These maxima can be used to partition the system into regions following the gradient flux (this would be like partitioning a set of mountains by the valleys) [12]. This partitioning gives rise to a set of non overlapping regions (or basins), labelled as Ω*i*. Their properties can be determined by integration over the region. Most commonly, the volume integration of the density, *ρ*, assigns a basin population, $\bar{N}({Ω}_{i})$:

(14) $$\bar{N}({Ω}_{i})=\int_{{Ω}_{i}}\rho (\vec{r})d\vec{r}$$

The properties of these basins have a chemical meaning inherited from the partition, so that the integration of the density over a basin associated with a bond maximum, can be understood as the bond charge.

## 3. Computational Details

Electronic structure calculations for a number of IV, III–V and II–VI solids with the diamond and zinc-blende-type structures were performed within the density-functional theory formalism. These compounds were selected to ensure the existence of a basin associated with the bond.

We have used the all-electron full potential linearised augmented-plane wave (FP-LAPW) approach as implemented in the ELK (for electrons in k-space) code [27]. The FP-LAPW method is among the most precise DFT-based methods for crystal structures [28]. It divides space into an interstitial region and non-overlapping muffin-tin (MT) spheres centered at the atomic sites. In the interstitial region, the basis set is described by plane-waves, whereas in the MT spheres, the basis set is described by radial solutions of the one particle Schrödinger equation and their energy derivatives multiplied by spherical harmonics. A high plane-wave cut-off *K_max_R_MT_* = 9, where *R_M__T_* is the smallest muffin-tin radius of the corresponding atomic species and *K_max_* is the maximum size of the reciprocal lattice vectors, guaranteed convergence in all the studied systems. Moreover, spherical harmonics within each atomic sphere were expanded up to the maximum angular quantum number *l_max_* = 14, to avoid discontinuities of the electron density and its derivatives at the muffin boundaries. Lattice parameters were set to the room temperature experimental values of the corresponding structures [29]. We have used both, the Perdew-Wang [30] local density approximation (LDA) and the Perdew-Burke-Ernzerhof [31] generalized gradient approximation (GGA) for the exchange-correlation functional. Brillouin-zone integrations were approximated using 8 *×* 8 *×* 8 Monkhorst-Pack grids, and the the self-consistent iteration process was repeated until the absolute change in total energy was lower than 1 *×* 10*^−^*^5^ hartrees.

In a second step, the topology of the ELF functions coming from the previous calculations is investigated using CRITIC, a code developed by some of the authors [32,33]. CRITIC is able not only to find ELF maxima and saddle points but it can also construct basins and integrate properties.

The training set has been chosen to lay in the shared-electron range. This characteristic is necessary so that there is a bond basin linkable to each pair of nearest neighbors in the crystal. In other words, it is possible to assign bonding parameters within the ELF framework.

### 3.1. Bond Length: R_B_, r_1_/r_2_

**Figure 2:**
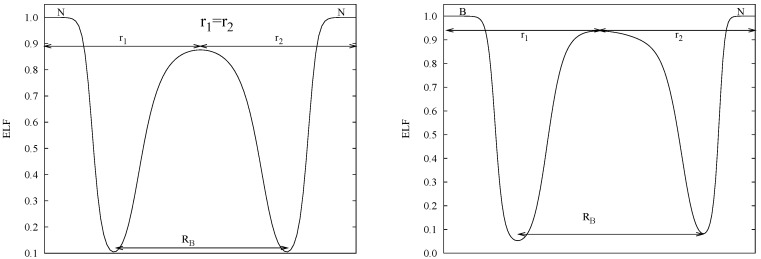
Definition of length-related bond charge model parameters, [*R_B_*, *r*_1_/*r*_2_], for a homonuclear molecule, N_2_ (**left**), and a heteronuclear molecule, BN (**right**).

The most common approach in the analysis of a bonding structure by means of the ELF approach is to use its 3D topology. However, the BCM uses a 1D definition of the chemical bond. Thus, we have devised an alternative route to obtain the BCM parameters from ELF. We have resorted to the 1D-ELF topology: ELF is analyzed along the straight line between pairs of atoms. In some cases, specially as going down the periodic table, this line might differ both from the AIM and ELF bonding lines, but it enables us to stick to the BCM formalism. On the one hand, the position of the bond charge within this length, *r*_1_/*r*_2_, (shifted from the center by the relative electronegativity of atoms) is determined by the local ELF maximum along the same direction. On the other hand, the distance between the two closest minima to the bond maximum along the internuclear direction provide the bond length, *R_B_* (See Figure 2).

## 4. Defining the Parameters from First Principles

ELF profiles along the internuclear line were constructed for each solid [14]. These profiles were then analyzed in order to find the position of the local maxima and minima, which enable the quantitative determination of *ab initio* *R_B_*, *r*_1_ and *r*_2_ values according to the previous definition. Results are collected in Table 1.

In homonuclear cases the local maximum is located in the center of *R_B_* and *r*_1_/*r*_2_ = 1.0 by definition (see N_2_ in Figure 2 (left)). Instead, in heteronuclear cases, the ELF profile is deformed and the maximum of the bond (or attractor) is no longer placed in the middle of *R_B_* (see BN in Figure 2 (right)). 

A similar value obtained for *R_B_* is obtained in all solids, with numbers clustering around 1 Å. This means that the size of the bond (at least along the interbonding direction) depends neither on the lattice parameter of the crystal (which spreads a range of more than 2.5 Å), nor on the two atoms involved in the bond.

Secondly, we observe that *r*_1_/*r*_2_ ≃ 1 ratios are obtained for the purely covalent families and those whose ∆χ ≃ 0. There is one remarkable outlier: BN shows *r*_1_/*r*_2_ ≃ 1 with ∆χ = 1. In order to understand this fact, an extra column has been added to Table 1 with the displacement of the attractor within the bond length *RB*, that is, taking away core contributions: $r_{1}^{\prime }=r_{1}-r_{A},r_{2}^{\prime }=r_{2}-r_{X}$.

This means that we now decompose the bond length into $R_{B}=r_{1}^{\prime }+r_{2}^{\prime }$, so that $R=r_{A}+r_{1}^{\prime }+r_{2}^{\prime }+r_{X}$. The physical meaning of these quantities is depicted in Figure 1 (right). This quantity enables us to understand that although the charge in BN is nearly at the same distance from B and from N, it is displaced within *R_B_*, as expected from their electronegativity difference (see Figure 2 (right)). This decomposition also helps to get rid of the core size in the comprehension of the system. Let’s take the case of BP: since P belongs to the second row and B to the first one, we have *r_X_*>> *r_A_*, so that eventhough the center of the bond is displaced within the bond towards P ($r_{1}^{\prime }>r_{2}^{\prime }$), once the core radii are taken into account, the distance relationship is inversed (*r*_2_ >* r*_1_).

### 4.1. Bond Charge: q_B_

Obtaining the charge associated with ELF entities through the integration over the corresponding basins might be considered in principle a straightforward and accurate method. However, for heavy elements, some ill-defined cases appear for integration, where the low symmetry of the basins and the appearance of spurious critical points at the muffin surfaces may lead to serious difficulties if we seek a quantitative analysis of its topology.

Based on pseudopotential principles, we assume that the core electronic distributions are practically unaltered by the environment, and thus *q_B_* values can be directly obtained by mere difference between the total and core number of electrons. Core values for the elements up to the 4th row involved in the solids under this study have been extracted from the ELF integrations gathered in [34]. Beyond the 4th row, calculations were perfomed for the atoms with the PBE functional [31] and the 6-31G* basis set, using the Gaussian 03 program [35]. Their core/valence charge, which is very stable to the level of calculation, was calculated with the TopMod program [36]. This assumption has the added value of providing extremely fast calculations. It has also been validated by comparison with the charges obtained by the integration over the corresponding 3D-basins with the CRITIC code [37]. For instance, integrated values for *q_B_* of 1.952 and 1.948 for C and BN, respectively, are in good agreement with the corresponding data in Table 1. They are also independent of the exchange-correlation functional used.

It has been shown that ELF charges lie very close to chemical intuition values [38]. This is further seen in Table 1. Nearly 2 electrons are recovered in all cases for the bonds, in agreement with their bond order of 1. This is different from the typical BCM charges obtained from fitting, which in many cases deviate from the expected values [17,18]. Furthermore, this image of *q_B_* correlating to the bond order does not agree with the definition of the *q_B_* parameter in the traditional model. The step forward that ELF provides concerns also the evaluation of the charge transfer (*δ*). In all cases (see Table 1), *δ* also shows reasonable values following the expected trends for the IV, III–V, and II–VI crystal families dictated by their electronegativity [23]. Keep in mind that *δ* is calculated with respect to valence configurations, so that for example carbon and silicon, having the same configuration, [34] give rise to *δ* = 0 in SiC.

This is an important characteristic of this Non Empirical New Bond Charge Model (NEWBCM), parameters are not only obtained *ab initio*, but they are closer to chemical expectations and recover/predict values in good agreement with experience (at difference with the initial BCM parameters).

## 5. Testing the Model: Response to Pressure

Since pressure can introduce significant changes in the stability of phases, it is most useful in the synthesis of novel phases and metastable materials. Pressure allows precise tuning of a fundamental parameter, the interatomic distance, which controls the electronic structure and virtually all the interatomic interactions that determine materials properties [39,40,41]. With pressure tuning, properties can often be more rapidly and cleanly optimized than with chemical tuning, which necessitates the synthesis of a large number of different materials and can induce disorder, phase separation, and other undesirable effects. Pressure tuning is therefore a useful tool in the search for new solid state materials with tailored properties.

### 5.1. Bulk Modulus

Among the description of the responses to pressure, compressibility (*κ*) or its inverse, the bulk modulus (*B*_0_), probably constitute the most widely used parameters. The inverse relationship between bulk modulus and volume has been widely explored [42,43,44,45], enabling to split the response of solids under pressure into local contributions (see for example [43,46,47,48]).

Following our BCM model, the valence charge is distributed along the chemical bonds, so that the compressibility of solids can be approximated to a good extent by the compressibility of the bonds, *κ_B_*, whereas cores are assumed to remain untouched: *κ* ≃ *κ_B_* [43,49]. The bulk modulus of the solid is then given by (see S.I. for the development):

(15) $$B_{0,B}={\left[V_{B}\left(\frac{\partial^{2}E_{B}}{\partial V_{B}^{2}}\;\right)\left(\frac{\partial V_{B}}{\partial V}\right)\right]}_{0}=\frac{Mq_{B}^{2}}{32\sqrt{3}R_{B}R^{3}}$$

where $V_{B}=\frac{1}{4}\left[V_{AXB_{4}}-(V_{A}+V_{X})\right]$ is the bond’s volume within the *AXB*_4_ unit.

**Figure 3:**
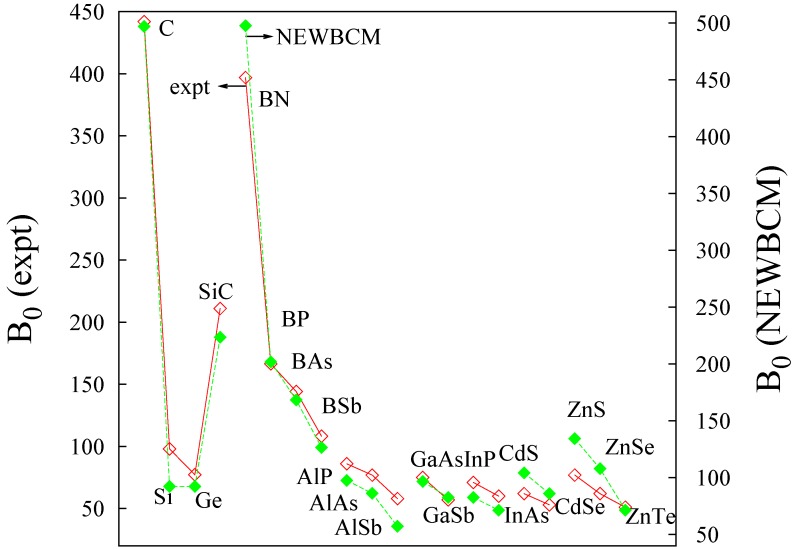
Experimental *vs*. non empirical new bond charge model (NEWBCM) bulk modulus (GPa).

Equation (15) for our IV, III–V and II–VI compounds is analyzed in Figure 3. It is worth highlighting that a very good agreement is obtained for all data. The slope for the bulk modulus is around 1 (1.14), and the offset is near 9 GPa (8.99 GPa) which departures from zero but it is not too large if we compare it with the range (up to 500 GPa) displayed by B0 in these compounds. The Pearson regression coefficient *R*2 is 0.9774. A similar agreement was obtained by Gilman *et al.* [1] in an enlightening analysis from empirical measures where the relationship was analyzed in terms of valence density (*i.e.*, *q_B_*/*V* ). The coherency between these two results can be perfectly understood from the analysis of Equation (15) if we take into account that $R=\frac{\sqrt{3}}{4}a$ and *V* = *a*^3^ for these crystals:

(16) $$B_{0,B}=\frac{2M}{9R_{B}}\frac{q_{B}^{2}}{V}$$

**Figure 4:**
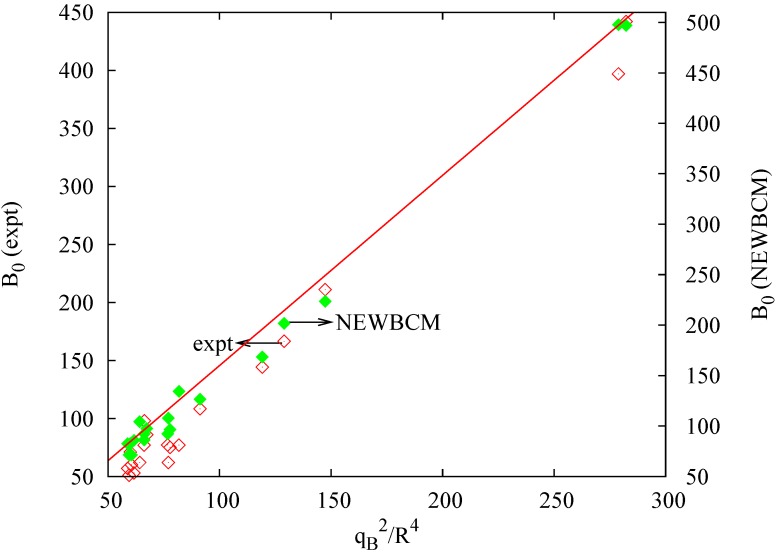
Experimental (empty symbols) and NEWBCM (full symbols) bulk moduli (in GPa) for IV, III–V and II–VI compounds. Bulk moduli in GPa, charge in electrons, *R* in atomic units.

In his fit, Gilman made use of the cell volume and an average valence occupation *q_B_* /*V* as would be expected from Lewis theory (*i.e.*, a default bond occupation *q_B_* = 2). Whereas the bond occupation has been found in our calculations to be very close to two, the dependence on the cell size can be improved by taking calculated bond lengths into account. Indeed, Equation (16) shows that the dependence on the lenght parameters should be *B*_0_ ∝ (*R_B_V*)^−1^ (*i.e.*, *B*_0_ ∝ (*R_B_R*^3^)^−1^, exponent nearly –4), whereas Gilman took *B*_0_ ∝ *V*^−1^ alone (exponent on *R* = –3). Indeed, a logarithmic fit of the *B*_0_ experimental data *vs.* *R* gives a slope of 3.8 (±0.1) (regression coefficient *R*^2^ = 0.9863) which explains the need to increase the length dependence from *R*^−3^ to *R*^−3^$R_{B}^{-1}$. As far as the bond charge is concerned, default occupations are very close to the values obtained with ELF, however, the quantitative values provided by electronic structure calculations enable us to obtain a better linear behavior. This is shown in Figure 4, where experimental and NEWBCM values have been included (in order to represent experimental values, the bond length dependence has been set to *R*^4^). It is interesting to note that, although calculations provide a better linear behavior (*R*^2^(NEWBCM) = 0.9927, *R*^2^(Exptal.) = 0.9830), the use of default or nominal (*q_B_* = 2) values already provides all the physics.

### 5.2. From Micro- to Macroscopic: Understanding Compressibility

Understanding the inherent relationship between (quantifiable) microscopic properties and macroscopic properties is the corner stone of material design.

We have shown that the concepts derived from ELF can be used to understand the microscopic origin of solids compressibility. If we analyze the dependencies of Equation (15), we see that the least compressible materials are found within the first period, where smaller interatomic distances, *R* ∝ *V*^1^*^/^*^3^, and bond lengths, *R_B_*, are found. While these results were already known from experience, the microscopic approach can also reveal new information. With this aim in mind, it is interesting to study the evolution of NEWBCM parameters (*r*_1_, *r*_2_, *R_B_* and *q_B_*) upon compression.

Let’s start with *R_B_*. Since *R_B_* = *νR* can be expressed as *R_B_* = *R−* *r_A_−* *r_X_*, and the core radii remain practicably untouched upon compression, a linear relationship necessarily holds for *R_B_* (which virtually subsumes all the changes in the cell parameter) with *R* for a given material.

Furthermore (and less intuitively), the linear relationship also holds for *r*_1_ and *r*_2_ with *R* at constant *δ*. According to Parr’s electronegativity approach [20], the electronegativity in the molecule *AX*, *χ_AX_*, can be expressed in terms of the radii of the isolated atoms, *r_A_* and *r_X_*, as: [19]

(17) $$\chi_{AX}=\frac{\chi_{A}r_{A}\;+\chi_{X}r_{X}}{R}$$

but also in terms of their properties in the molecule *AX*:

(18) $$\chi_{AX}=K\frac{q+\delta }{r_{1}}$$

where *K* is a constant.

If we combine Equations (17) and (18) and reorganise, we see that:

(19) $$r_{1}=\frac{K(q+\delta )}{r{\;}_{A}\chi_{A}+r_{X}\chi_{X}}R=\alpha R$$

where *K*, *q*, *r_A_*, *r_X_*, *χ_A_* and *χ_X_* are constants which only depend on the system, so that *α* itsef is a constant as long as *δ* remains constant. In other words, *r*_1_ will scale linearly with the interatomic distance, as long as the charge transfer does not change upon pressurization. Of course, similar equations apply to *r*_2_, with *r*_2_ = *βR*.

**Figure 5:**
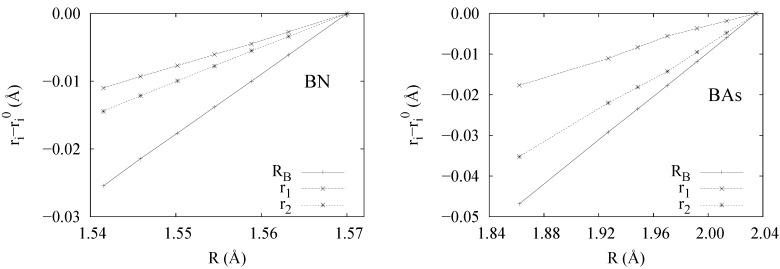
Evolution of the geometrical parameters, *r*_1_, *r*_2_ and *R_B_*, upon compression of the interatomic distance *R* for BN (**left**) and BAs (**right**). Data relative to the corresponding equilibrium values denoted by a 0 superscript. All numbers in Å.

In order to check this hypothesis, we have repeated our DFT-ELF procedure upon compression of the zero pressure reference cell (no optimization needed due to the symmetry of the cell). Two prototypical examples of covalent (BAs, ∆*_χ_* = 0.14) and polar (BN, ∆*_χ_* = 1) solids were chosen to compare their corresonding compression with respect to their bonding type. Figure 5 collects the results upon compression for *r*_1_, *r*_2_ and *R_B_*. Negligible deviations from linearity are found in both cases, leading to three main conclusions:

- *R_B_* is the quantity showing the greatest compression of the model since it represents the valence size, i.e. the chemically sensitive part as pressure is applied [43,49].
- *α*, *β* and *ν* are constant upon compression for the various types of bonding considered. Further analysis shows that *α*, *β* and *ν* can certainly be related to hardness, since they determine which ion compresses the most. Indeed a look at Figure 5 shows that in the case of BN, where the hardness of atoms is more similar, *r*_1_ and *r*_2_ compress at similar rates, whereas in BAs, the compression of As overcomes that of B.
- As a consequence of the previous observation and from Equation (19), we can consider that the charge transfer is also constant upon compression for the ranges considered.

If we analyze these constants, it is observed that the hardest materials, diamond and BN, not only have the smallest *R* and the smallest *R_B _*, but also the highest *ν* = *R_B _*/*R* ratio (see Table 1).

The size of the parameters *r*_1_ and *r*_2_ (or *α* and *β*) also plays an important role. Since these parameters are related to the relative hardness of atoms, the smaller *α* and *β* are, the less compressible the whole system becomes. What is most important, both parameters should be similarly small. Otherwise, the softest ion will subsume the pressure. Ideally, for a given *r*_1_, the compressibility is minimized when the relationship *α* = *β* is fulfilled. This condition is equivalent to locating the bond position in the middle of both atoms (see Figure 5 (left)).

These dependencies enable us to establish new relationships for materials with low compressibility, directly derived from microscopic conditions. From Table 1 we see that there are two cases which deserve our attention as far as their *r*_1_/*r*_2_ ratio is concerned: diamond and BN represent the two different ways of achieving *α* = *β* (*i.e.*, *r*_1_ = *r*_2_):

- Perfectly covalent case (diamond): in this case *A* = *X* (or ∆*_χ_* = 0), so that all lengths are the same.
- Polar case (BN): in this case *A* ≠ *X* (∆*_χ_* ≠ 0). However, a relationship holds between the core and the bond location displacement. It is found that the cores differential size, *ϵ* = *r_X_−* *r_A_* for *ϵ* > 0 is equivalent to the displacement of the bond location from the center toward the smaller atom *A*, $r_{1}^{\prime }=r_{2}^{\prime }+\epsilon$ (remember that *r*_1_ = $r_{1}^{\prime }$ + *r_A_* and *r*_2_ = $r_{2}^{\prime }$ + *r_X_*). In BN, a good combination of atomic sizes and polarity compensate to provide *r*_1_/*r*_2_ = 1.03.

## 6. Summary and Conclusions

We have introduced the Electron Localization Function as the source of the necessary parameters for the analysis of the Bond Charge Model in solid state applications, named NEWBCM. The availability of charges and bond location has enabled us to develop a new BCM-based model in which, parameters are not only obtained *ab initio*, but they are closer to chemical intuition and recover good agreement with experience.

The ability of NEWBCM to describe compressibility trends among these crystal families has been exploited in order to settle the directions for novel superhard materials design. Some of these hints are not related to macroscopic properties but to microscopic ones. Thus, quantitative relationships between electronic structure and macroscopic properties, such as compressibility, can now be proposed.

The micro-macroscopic properties that guide the quest for superhard materials can be summarized as follows: (i) Small atoms; (ii) Small bond; (iii) High *ν*; (iv) Hard atoms; and (v) Atoms of similar hardness. Although most of these guidelines are related (e.g., (i) and (iv)) and extremely intuitive, some of them, such as (iii) would not be deduced at first glance, and provide useful quantitative insight into the search and prediction of novel superhard materials from microscopic grounds.

As an example, it has been shown that the hardest compounds within our study set, diamond and BN, both follow these principles, and more specifically, both show an *r*_1_/*r*_2_ ratio very close to one in spite of belonging to two very different bonding types (covalent and very polar, respectively). These conditions thus unite their different macroscopic properties into one relevant microscopic condition for superhardness.

Although this model is only applicable to zinc-blende types of structures, work is in progress to extend it to other structural types. This would, for example, enable us to understand the driving forces for chemical changes in phase transitions.
